# Study protocol of the Aerobic exercise and CogniTIVe functioning in women with breAsT cancEr (ACTIVATE) trial: a two-arm, two-centre randomized controlled trial

**DOI:** 10.1186/s12885-020-07196-3

**Published:** 2020-07-31

**Authors:** Jennifer Brunet, Meagan Barrett-Bernstein, Kendra Zadravec, Monica Taljaard, Nathalie LeVasseur, Amirrtha Srikanthan, Kelcey A. Bland, Barbara Collins, Julia W. Y. Kam, Todd C. Handy, Sherri Hayden, Christine Simmons, Andra M. Smith, Naznin Virji-Babul, Kristin L. Campbell

**Affiliations:** 1 School of Human Kinetics, Faculty of Health Sciences, University of Ottawa, 125 University Private, Montpetit Hall, Ottawa, ON K1N 6N5 Canada; 2 Rehabiliation Sciences Graduate Program, Faculty of Medicine, University of British Columbia, T114-2211 Wesbrook Mall, Vancouver, BC V6T 1Z7 Canada; 3 The Ottawa Hospital Research Institute, Clinical Epidemiology Program, 501 Smyth Road, Ottawa, ON K1H 8L6 Canada; 4 British Columbia Cancer Agency, Vancouver, 600 West 10th Avenue, Vancouver, BC V5Z 4E6 Canada; 5 Department of Medicine, The Ottawa Hospital, 501 Smyth Road, Ottawa, ON K1H 8L6 Canada; 6 Mary MacKillop Institute for Health Research, Australian Catholic University, Level 5, 215 Spring Street, Melbourne, VIC 3000 Australia; 7 School of Psychology, Faculty of Social Sciences, University of Ottawa, 136 Jean-Jacques Lussier, Vanier Hall, Ottawa, ON K1N 6N5 Canada; 8 Department of Psychology & Hotchkiss Brain Institute, University of Calgary, 2500 Campus Drive, Calgary, AB T2N 1N4 Canada; 9 Department of Psychology, University of British Columbia, 3406-2136 West Mall, Vancouver, BC V6T 1Z4 Canada; 10 Division of Neurology/Faculty of Medicine, University of British Columbia, P213-2211 Westbrook Mall, Vancouver, BC V6T 2B5 Canada; 11 Department of Physical Therapy, Faculty of Medicine, University of British Columbia, 2215 Westbrook Mall, Vancouver, BC V6T 1Z3 Canada

**Keywords:** Randomized controlled trial, Chemotherapy-related cognitive changes, Chemo-brain, Aerobic exercise, Breast neoplasm

## Abstract

**Background:**

Up to 75% of women diagnosed with breast cancer report chemotherapy-related cognitive changes (CRCC) during treatment, including decreased memory, attention, and processing speed. Though CRCC negatively impacts everyday functioning and reduces overall quality of life in women diagnosed with breast cancer, effective interventions to prevent and/or manage CRCC are elusive. Consequently, women seldom receive advice on how to prevent or manage CRCC. Aerobic exercise is associated with improved cognitive functioning in healthy older adults and adults with cognitive impairments. Accordingly, it holds promise as an intervention to prevent and/or manage CRCC. However, evidence from randomized controlled trials (RCTs) supporting a beneficial effect of aerobic exercise on CRCC is limited. The primary aim of the ACTIVATE trial is to evaluate the impact of supervised aerobic exercise on CRCC in women receiving chemotherapy for breast cancer.

**Methods:**

The ACTIVATE trial is a two-arm, two-centre RCT. Women diagnosed with stage I-III breast cancer and awaiting neo-adjuvant or adjuvant chemotherapy are recruited from hospitals in Ottawa (Ontario) and Vancouver (British Columbia), Canada. Recruits are randomized to the intervention group (aerobic exercise during chemotherapy) or the wait-list control group (usual care during chemotherapy and aerobic exercise post-chemotherapy). The primary outcome is cognitive functioning as measured by a composite cognitive summary score (COGSUM) of several neuropsychological tests. Secondary outcomes are self-reported cognitive functioning, quality of life, and brain structure and functioning (measured by magnetic resonance imaging (MRI)/functional MRI and electroencephalography). Assessments take place pre-chemotherapy (pre-intervention), mid-way through chemotherapy (mid-intervention/mid-wait period), end of chemotherapy (post-intervention/post-wait period; primary endpoint), 16-weeks post-chemotherapy, and at 1-year post-baseline.

**Discussion:**

Aerobic exercise is a promising intervention for preventing and/or managing CRCC and enhancing quality of life among women diagnosed with breast cancer. The ACTIVATE trial tests several novel hypotheses, including that aerobic exercise can prevent and/or mitigate CRCC and that this effect is mediated by the timing of intervention delivery (i.e., during versus post-chemotherapy). Findings may support prescribing exercise during (or post-) chemotherapy for breast cancer and elucidate the potential role of aerobic exercise as a management strategy for CRCC in women with early-stage breast cancer.

**Trial registration:**

The trial was registered with the ClinicalTrials.gov database (NCT03277898) on September 11, 2017.

## Background

The current 5-year survival rate for breast cancer is 88% in Canada, emphasizing the need to address the adverse long-term effects of breast cancer treatment [1–4]. Up to 75% of women who receive chemotherapy for breast cancer report a decreased ability to remember, concentrate, and/or think both in the short- and long-term [5–7]. Results of meta-analyses also indicate that women who receive chemotherapy for breast cancer perform more poorly on neuropsychological tests assessing executive functioning, working memory, processing speed, spatial ability, and language/verbal ability when compared to women diagnosed with breast cancer who have not received chemotherapy or to controls without a history of cancer [8–11]. Chemotherapy-related cognitive changes (CRCC) typically manifest during treatment and can persist for many years post-chemotherapy [12–15]. Declines in cognitive functioning, even when minor, can lead to significant psychological distress and profoundly impact daily functioning and quality of life [14, 16–19].

Aerobic exercise has been shown to improve cognitive functioning in older adults [20–23] and in those with mild cognitive impairments [24, 25]. Specifically, improvements in executive functioning, working memory, attention, visuospatial memory, and processing speed are consistently reported by those who engage in aerobic exercise [26]. There is also emerging evidence that aerobic exercise can alter both brain structure and functioning [24, 25, 27, 28]. Animal and human research supports several biological mechanisms and neural changes for the effect of aerobic exercise on cognitive functioning, including decreased systemic inflammation and oxidative stress, enhanced plasticity of the brain, increased levels of brain-derived neurotropic factor, and improved cerebral blood flow and hemoglobin levels [20, 29]. In animal research, Fardell et al. [30] evaluated the effect of chemotherapy on cognitive functioning in rodents and assessed whether exercise could mitigate associated cognitive deficits. When compared to untreated control rodents, rodents treated with chemotherapy performed significantly worse on memory tasks, and specifically those tasks which strongly activated the hippocampus. However, among the rodents treated with chemotherapy, those randomized to a cage that allowed unlimited access to a running wheel displayed preserved cognitive functioning, particularly in terms of novel object recognition and spatial reference memory, compared to those randomized to a standard cage. These findings are consistent with those of Winocur et al. [31] who tested the effects of exercise on cognitive task performance and hippocampal neurogenesis in rodents following administration of chemotherapy. The authors reported that hippocampal neurogenesis was not suppressed in rodents receiving chemotherapy and housed in a cage that allowed unlimited access to a running wheel, and that cognitive performance was similar when compared to controls.

In human research, observational studies in women diagnosed with breast cancer who previously received chemotherapy show better cognitive functioning in those with higher levels of aerobic exercise, as measured by self-report [32–34], accelerometers [34–37], and aerobic fitness [35, 38, 39]. However, the potential of exercise to improve CRCC is unclear. A recent systematic review of 29 published randomized controlled trials (RCTs) found limited evidence for the benefit of exercise compared to usual care on CRCC in adults diagnosed with cancer, including those diagnosed with breast cancer [40]. Three RCTs in women receiving chemotherapy for breast cancer reported a statistically significant effect of aerobic exercise on CRCC, as measured by performance-based neuropsychological tests (i.e., objective measures [41] and self-report questionnaires [41–43]). In women who had completed chemotherapy for breast cancer, only one small proof-of-concept trial to date reported a statistically significant benefit of aerobic exercise on CRCC, as measured by performance-based neuropsychological tests [44]. Importantly, in their review, Campbell et al. [40] noted that many of the RCTs identified had evaluated cognitive functioning as a secondary outcome, often using a single item or subscale of a questionnaire assessing fatigue or quality of life. Cognitive functioning was evaluated as the primary outcome in only two RCTs in women diagnosed with breast cancer: one in women receiving chemotherapy [45] and one in women who had completed chemotherapy [44]. There is a clear need for an adequately powered RCT in women diagnosed with breast cancer to test the effect of aerobic exercise on CRCC, and the timing of such exercise (i.e., during versus post-chemotherapy), using both objective and self-report outcome measures of cognitive functioning.

A parallel two-arm RCT will be undertaken to determine if aerobic exercise is an effective strategy to prevent and/or mitigate CRCC and its impact on quality of life among women who receive chemotherapy for breast cancer. The primary objective of the Aerobic exercise and CogniTIVe functioning in women with breAsT cancEr (ACTIVATE) trial is to test the effect of an aerobic exercise intervention initiated during chemotherapy (EX) compared to a usual care wait-list control group (exercise initiated post-chemotherapy; UC) on objectively measured cognitive functioning in women who receive chemotherapy for breast cancer. Secondary objectives are to: (1) test the effects of EX compared to UC on (a) self-reported cognitive functioning, (b) global and regional measures of brain structure and functioning with magnetic resonance imaging (MRI) and functional MRI (fMRI) focussing on areas underlying attention and working memory, and (c) overall global and regional brain network organization and neural functioning in areas underlying attention and working memory using electroencephalography (EEG), and; (2) assess if the timing of the intervention (i.e., during versus post-chemotherapy) moderates the effects of exercise. It is hypothesized that: (1) the EX group will perform better on neuropsychological tests (primary outcome) than the UC group post-chemotherapy (primary endpoint) and at other timepoints; (2) the EX group will maintain pre-chemotherapy brain structure and functioning post-chemotherapy and at other timepoints whereas the UC group will have significant changes in both, and; (3) the EX group will self-report better cognitive functioning and psychosocial outcomes (i.e., quality of life, psychological health, cancer-related fatigue) post-chemotherapy and at other timepoints compared to the UC group.

## Methods/design

This manuscript was written in accordance with the SPIRIT guidelines ([46]; SPIRIT Checklist provided in Additional file 1). Ethics approval was granted by the research ethics boards at the University of Ottawa (Ottawa, ON) and the University of British Columbia (Vancouver, BC), as well as relevant hospital research ethics committees (i.e., Ottawa Health Science Network, the Royal Ottawa Mental Health Centre, and the BC Cancer research ethics boards). This trial was registered with the ClinicalTrials.gov database (NCT03277898; September 11, 2017).

### Study design

**Fig. 1:**
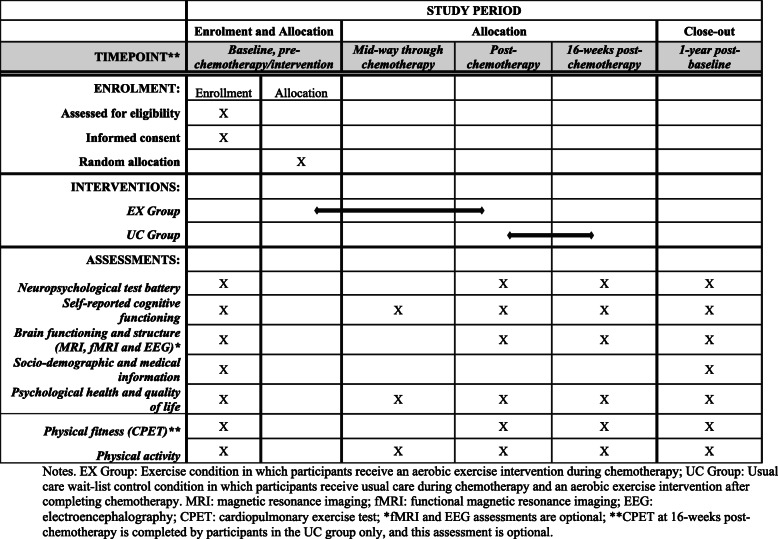
SPIRIT flow diagram for the schedule of enrollment, interventions, and assessments for the ACTIVATE trial

**Fig. 2:**
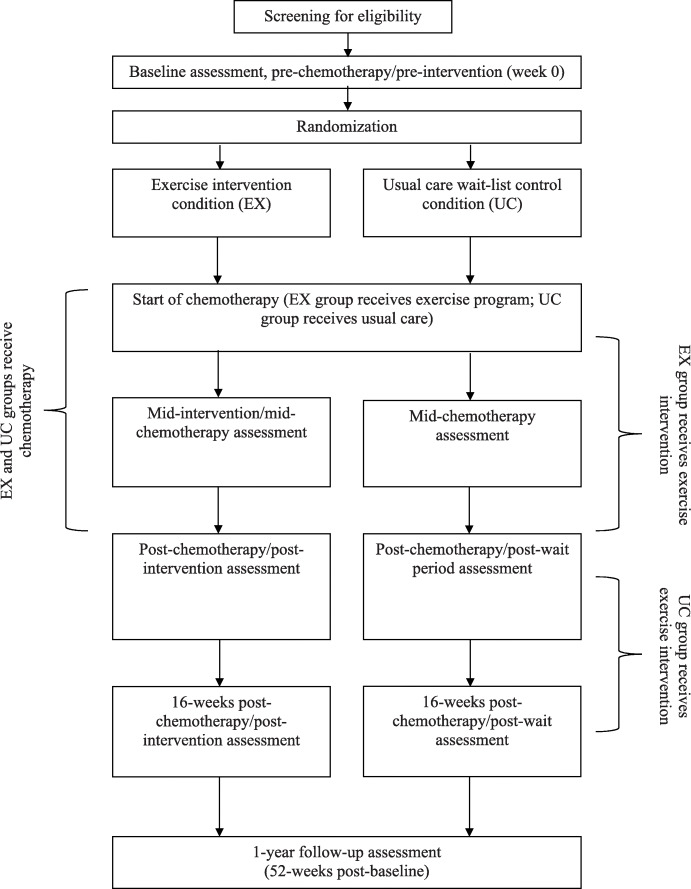
The ACTIVATE trial design

The ACTIVATE trial is a two-arm, two-centre, single-blinded parallel group RCT. After completing a baseline (pre-chemotherapy) assessment, recruited women are randomized to one of two groups: (1) exercise condition in which they receive an aerobic exercise intervention during chemotherapy (EX), or; (2) usual care wait-list control condition in which they receive usual care during chemotherapy and an aerobic exercise intervention after completing chemotherapy (UC). Additional assessments take place mid-way through chemotherapy (mid-intervention/mid-wait period), post-chemotherapy (post-intervention/post-wait period), 16-weeks post-chemotherapy (first follow-up), and at 1-year post-baseline (second follow-up). Figures 1 and 2 summarize the ACTIVATE trial design and the process of randomization, group allocation, and assessment timepoints.

### Recruitment and procedures

Potential participants are recruited from BC Cancer and The Ottawa Hospital (TOH) using several recruitment strategies. First, healthcare providers identify potentially eligible patients and introduce the study to them, asking those who are interested in participating for their permission to be contacted by study staff. Healthcare providers are advised to refer only those patients whom they medically clear to participate in the intervention and to provide study staff the name and phone number of potentially eligible/interested patients via email or by filling out a study recruitment form. Study staff then contact patients to review their eligibility and obtain their informed consent to participate in the study (if they are eligible and interested). Potential participants are also recruited via: (1) posters placed in waiting rooms and examination rooms at BC Cancer (Vancouver/Surrey sites) and at TOH and Irving Greenburg Family Cancer Centre (which is a satellite of TOH Cancer Centre; Ottawa site); (2) advertisements posted on the co-principal investigators’ research lab websites, and; (3) word of mouth. Interested women are asked to contact study staff via phone or email for more information.

### Participant eligibility

Women are eligible to participate if they: (1) are 19–70 years of age; (2) have been diagnosed with stage I-III (i.e., non-metastatic) breast cancer; (3) are scheduled to receive adjuvant or neo-adjuvant chemotherapy; (4) are able to speak and understand English, and; (5) have approval from their medical oncologist to participate in the exercise intervention. They must also complete a cardiopulmonary exercise test (CPET) prior to randomization and be cleared by a cardiologist. Ineligibility criteria include: (1) previous exposure to chemotherapy or radiation therapy; (2) score ≤ 23 on the Montreal Cognitive Assessment (MoCA [47, 48]) during screening; (3) diagnosis of a severe anxiety or mood disorder (e.g., major depressive disorder) by a medical professionalwithin the past year; (4) traumatic brain injury or concussion with residual symptoms (e.g., dizziness, headaches, loss of concentration) at the time of screening; (5) diagnosis of a substance use disorder (e.g., alcohol, narcotics) by a medical professional; (6) meet physical activity guidelines for general health of 150 min of moderate-to-vigorous-intensity aerobic exercise per week [49–51] in the 3 months prior to enrollment; (7) body mass index ≥45 kg/m^2^, and/or; (8) mobility issues that require a mobility aid or an injury/illness (e.g., orthopedic injury, severe arthritis) that prevents exercise on a bike, treadmill, or elliptical.

#### Additional inclusion/exclusion criteria for MRI/fMRI and EEG

During the initial screening process, potential participants are asked if they would also be interested in participating in an additional optional assessment consisting of an MRI/fMIR and/or EEG (EEG assessment is at the Vancouver site only). The MRI/fMRI and EEG are performed at three timepoints: baseline (before chemotherapy; pre-intervention), post-chemotherapy (post-intervention/post-wait period), and at 1-year post-baseline. The following additional exclusion criteria apply to the MRI/fMIR assessment: (1) left-handedness (due to language lateralization in right-handers); (2) metal implants (e.g., pacemaker) or metal dental work aside from fillings (as these are not compatible with MRI/fMRI); (3) current breast tissue expanders (as these are not compatible with MRI/fMRI); (4) claustrophobia; (5) poor eyesight not correctable with contact lenses or MRI/fMRI safety goggles (as participants must be able to view the stimuli presented in the scanner), and; (6) lower back pain that would preclude lying relatively still for 1 h. There are no additional criteria for participating in the EEG assessment besides the main trial criteria.

#### Screening procedure and informed consent

Study staff perform an initial screening of women by phone to ensure they meet the main eligibility criteria. If women are deemed eligible following the initial screening by phone, in-person assessments are scheduled to administer a CPET and the MoCA [48], which is a brief 30-point test used to measure cognitive impairments. The CPET and MoCA are used to determine women’s final eligibility and are administered prior to performing any study-related activities. Patients who score ≥ 24 on the MoCA are invited to read and sign the informed consent form (prior to performing a CPET).

#### Randomization

After providing informed consent, completing baseline assessments, and receiving final medical clearance from a cardiologist, participants are randomized to the EX or UC condition in a 1:1 ratio, stratified by site (i.e., Vancouver versus Ottawa) and menopausal status (i.e., pre/peri-menopausal versus menopausal) at breast cancer diagnosis. The allocation sequence is computer-generated by a statistician at TOH Methods Center, and randomization is performed by study staff who log onto a secure server website to obtain the next allocation.

#### Blinding

Participants and study staff are unaware of group allocation during baseline assessments since randomization is performed after participants have completed their baseline assessments. Following randomization, a single-blind procedure is followed whereby study staff performing assessments, as well as the statistician analyzing the data, are blinded to group allocation.

#### Sample size

A power calculation was performed to detect changes in cognitive functioning (as measured by a composite cognitive summary score (COGSUM; developed by Collins et al. [52–55]). Using a minimum clinically important difference of 0.4 standard deviation units between EX versus UC at post-chemotherapy (i.e., post-intervention/post-wait period; primary endpoint), a total sample size of 74 women is needed to achieve 80% power using an analysis of covariance (ANCOVA) at a two-sided 5% level of significance. The correlation with the baseline measure was assumed to be 0.8. To account for a potential dropout rate of 10%, a sample of 84 participants (42 per condition) will be recruited.

#### The ACTIVATE intervention (EX condition)

The ACTIVATE intervention is delivered concurrently with participants’ chemotherapy regimen (starting up to 1 week before participants’ chemotherapy start date and lasting approximately 12–24 weeks for those in the EX group). Tables 1 and 2 provide an overview of the progressive aerobic exercise prescription. The intervention is based on prior protocols used by the study team for women receiving treatment for breast cancer or who have completed treatment for breast cancer [56–59]. Participants complete three supervised aerobic exercise sessions each week at the Breast Cancer Training Center or at the Chan Gunn Pavilion on the University of British Columbia campus for the Vancouver site, or at the University of Ottawa Behavioural and Metabolic Research Unit for the Ottawa site. Supervision of the exercise sessions is provided by trained exercise professionals who have a valid CPR-C certification and experience supervising exercise in chronic disease populations.

**Table 1:** Details of supervised and home-based exercise prescription for participants on a 3-week chemotherapy cycle protocol

Cycle	Week	Supervised Program			Home-based Program		
		Freq.	Duration (minutes)	Intensity (% HRR)	Freq.	Duration (minutes)	Intensity (RPE)
1	1	3	20–25	50–55^a^, 55–60	0	0	0
	2	3	30	55–60	0	0	0
	3	3	25–30	60–65	1	15–20	12–13
2	4	3	30–35	50–55^a^, 60–65	1	15–20	12–13
	5	3	25–35	60–65, VT	1	15–20	12–13
	6	3	25–35	60–65, VT	1	20–30	12–13
3	7	3	35–40	55–60^a^, 60–65	1	20–30	12–13
	8	3	25–35	65–70, VT	1	20–30	12–13
	9	3	25–35	65–70, VT	1	20–30	12–13
4	10	3	35–40	55–60^a^, 65–70	1	20–30	12–13
	11	3	20–35	65–70, HIIT	1	20–30	12–13
	12	3	20–40	65–70, HIIT	1	20–30	12–13
5	13	3	40	55–60^a^, 65–70	1	20–30	12–13
	14	3	20–35	70–75, VT/HIIT^b^	1	20–30	12–13
	15	3	20–35	70–75, VT/HIIT^b^	1	20–30	12–13
6	16	3	40	55–60^a^, 65–70	1	20–30	12–13
	17	3	20–35	70–75, VT/HIIT^b^	1	20–30	12–13
	18	3	20–35	70–75, VT/HIIT^b^	1	20–30	12–13
7	19	3	40	55–60^a^, 65–70	1	20–30	12–13
	20	3	20–35	70–75, VT/HIIT^b^	1	20–30	12–13
	21	3	20–35	70–75, VT/HIIT^b^	1	20–30	12–13
8	22	3	40	55–60^a^, 65–70	1	20–30	12–13
	23	3	20–35	70–75, VT/HIIT^b^	1	20–30	12–13
	24	3	20–35	70–75, VT/HIIT^b^	1	20–30	12–13

*Freq.* Frequency, *HIIT* high-intensity interval training session (10–16 bouts of 30 s at 100% peak workload and 1–2 min of active recovery), *HRR* Heart rate reserve, *RPE* Rating of perceived exertion, *VT* ventilatory threshold session (4 bouts of 5 min at VT and 3 min of active recovery)

^a^The first exercise session immediately after chemotherapy infusion is prescribed at 50–55% HRR (first two cycles) and 50–60% HRR (remaining cycles) (i.e., 5–10% reduction in workload). ^b^After Cycle 5, VT and HIIT sessions are prescribed based on participants' preference. Participants perform a 5-min warm-up before and 5-min cool-down after each exercise session

**Table 2:** Details of supervised and home-based exercise prescription for participants on a 2-week chemotherapy cycle protocol

Cycle	Week	Supervised Program			Home-based Program		
		Freq.	Duration (minutes)	Intensity (% HRR)	Freq.	Duration (minutes)	Intensity (RPE)
1	1	3	20–25	50–55^a^, 55–60	0	0	0
	2	3	25–30	55–65	0	0	0
2	3	3	25–35	50–55^a^, 60–65	1	15–20	12–13
	4	3	25–35	60–65, VT	1	15–20	12–13
3	5	3	35–40	55–60^a^, 60–65	1	15–20	12–13
	6	3	25–35	65–70, HIIT	1	20–30	12–13
4	7	3	35–40	55–60^a^, 65–70	1	20–30	12–13
	8	3	20–40	65–70, HIIT	1	20–30	12–13
5	9	3	40	55–60^a^, 65–70	1	20–30	12–13
	10	3	20–35	70–75, VT/HIIT^b^	1	20–30	12–13
6	11	3	40	55–60^a^, 65–70	1	20–30	12–13
	12	3	20–35	70–75, VT/HIIT^b^	1	20–30	12–13
7	13	3	40	55–60^a^, 65–70	1	20–30	12–13
	14	3	20–35	70–75, VT/HIIT^b^	1	20–30	12–13
8	15	3	40	55–60^a^, 65–70	1	20–30	12–13
	16	3	25–35	70–75, VT/HIIT^b^	1	20–30	12–13

*Freq.* Frequency, *HIIT* high-intensity interval training session (10–16 bouts of 30 s at 100% peak workload and 1–2 min of active recovery), *HRR* Heart rate reserve, *RPE* Rating of perceived exertion, *VT* ventilatory threshold session (4 bouts of 5 min at VT and 3 min of active recovery).

^a^The first exercise session immediately after chemotherapy infusion is prescribed at 50–55% HRR (first two cycles) and 50–60% HRR (remaining cycles) (i.e., 5–10% reduction in workload). ^b^After Cycle 5, VT and HIIT sessions are prescribed based on participants' preference. Participants perform a 5-min warm-up before and 5-min cool-down after each exercise session

Participants follow a “chemotherapy-periodized”, non-linear aerobic exercise training protocol. Periodization is an organizational approach that can be applied to aerobic exercise and is frequently used in sport performance involving short cycles or “periods” of systematic variation in training specificity, intensity, and volume [60]. The study team has previously shown that this type of training can be modified for women diagnosed with breast cancer receiving chemotherapy and is associated with higher supervised exercise session attendance compared to a standard linear aerobic exercise prescription [59]. For the current trial, periods are matched to the length of participants’ chemotherapy protocol. For the first period, corresponding to 1 week (for participants on a 2-week chemotherapy protocol) and 2 weeks (for participants on a 3-week chemotherapy protocol), participants perform moderate continuous aerobic exercise training at 50–75% heart rate reserve (HRR) after each chemotherapy infusion. The first exercise session immediately following each chemotherapy infusion isprescribed at 50–60% HRR, when treatment symptoms are expected to be worse. For the weeks following the first period and prior to the next chemotherapy infusion, aerobic exercise intensity isprogressively increased and includes a combination of maximal steady state exercise training (ventilatory threshold) and high-intensity interval training (based on maximum heart rate and workload achieved during the CPET). Participants are provided with individualized target heart rates (all calculated based on baseline CPET data) and are provided with a Polar heart rate monitor (Polar Electro Inc., Lake Success, NY) during the supervised sessions to monitor their heart rate. During the supervised exercise sessions, the trained exercise professionals record the type (e.g., treadmill, elliptical, bike) and duration of exercise performed, as well as participants’ average heart rate and rating of perceived exertion using the 6–20 Borg scale [61]. Both sites have treadmills, stationary bikes, and elliptical machines, and participants are encouraged to use at least two different typesof exercise each week to help reduce the risk of overuse injury. Home-based exercise is introduced in week three of the intervention. Participants are asked to participate in at least one session per week (lasting 15–20 min for the first 3 weeks of the home-based program and 20–30 min for the remainder of the program) of an aerobic exercise of their choosing (e.g., walking) to help them progress towards meeting the current physical activity guidelines for cancer survivors [62]. Participants are also encouraged to complete an additional home-based session when a supervised exercise session is missed.

#### Comparison condition: usual care wait-list control (UC)

Participants who are allocated to the UC condition receive the ACTIVATE intervention for approximately 12 weeks following their last chemotherapy infusion. They are advised to continue with their regular activities of daily living during chemotherapy. No exercise restrictions are made.

### Data collection

#### Primary outcome

##### Objective assessment of cognitive functioning

At four of the five study timepoints (i.e., baseline (pre-intervention), end of chemotherapy (post-intervention/post-wait period; primary endpoint), 16-weeks post-chemotherapy (first follow-up), and at 1-year post-baseline (second follow-up)), participants complete a neuropsychological test battery that is used to compute a composite cognitive summary score (COGSUM). The battery covers verbal and visual memory, attention/working memory, and processing speed, and was chosen because these cognitive domains have been shown to be sensitive to adverse effects of chemotherapy in previous studies [54]. Moreover, the specific tests were chosen because they have accounted for most of the between-group variance observed with a larger test battery [54]. The order of tests is as follows: Hopkins Verbal Learning Test Revised (verbal memory [63]), Brief Visuospatial Memory Test Revised (visual memory [64]), Weschler Adult Intelligence Scale-IV (WAIS-IV) Digit-Symbol Coding (processing speed [65]), WAIS-IV Letter-Number-Sequencing (working memory [65]), Auditory Consonant Trigrams Test (working memory [66]), Controlled Oral Word Association Test (working memory [67]), and Trail Making Test A and B (processing speed and cognitive flexibility/task switching capacity [68]). To reduce practice effects, different testing forms are used at each timepoint, with the exception of the WAIS-IV tests, Controlled Oral Word Association Test, and Trail Making Tests A and B, where alternate forms are not available.

#### Secondary outcomes

##### Self-reported cognitive functioning

At each of the five study timepoints, participants complete self-report questionnaires assessing cognitive functioning and its impact on quality of life. These include the Functional Assessment of Cancer Therapy-Cognitive Function (FACT-Cog) Version 3 [69, 70] and the Patient-Reported Outcomes Measurement Information System Applied Cognition short form [71–73].

##### Brain functioning and structure (MRI/fMRI and EEG)

Optional neuroimaging (i.e., MRI/fMRI) and EEG are completed in a sub-set of interested and eligible participants at three timepoints: baseline (pre-intervention), end of chemotherapy (post-intervention/post-wait period; primary endpoint), and at 1-year post-baseline (second follow-up). The MRI assessment is a 60-min scan comprised of a high-resolution structural scan, a resting state fMRI procedure, a diffusion tensor imaging sequence, and two fMRI tasks designed to measure working memory (i.e., Letter N-Back Task [74] and recognition memory (i.e., Word List Recognition Task [75]). These tasks were selected as physiological differences (e.g., brain activity) have been observed from pre- to post-chemotherapy during these tasks in women treated for breast cancer [75, 76]. Imaging takes place with a Siemens Biograph 3 Tesla Magnetom MR Scanner equipped with a 12-channel head coil. All data will be analysed with SPM12 and FSL.

The EEG assessment (Vancouver site only) is performed using a 64-channel Hydrogel Geodesic SensorNets (EGI, Eugene, OR). Five minutes of resting data are collected while participants have their eyes closed. Participants then perform a 25-min modified visual sustained attention to response task (SART) while their EEG is recorded, where they are asked to respond to a serial sequence of digits and withhold responses to any letters that appear [77–80]. EEG results are recorded and amplified using the Net Amps 300 amplifier at a sampling rate of 250 Hz. Scalp electrode impedances are under 50 kΩ. The signals are referenced to the vertex (Cz) and filtered from 4 to 40 Hz. A notch filter at 60 Hz is applied. The EEG signals will be analyzed offline using Brain Electrical Source Analysis (BESA; MEGIS Software GmbH). An automated artifact scan available by BESA is performed for extracting motion and excessive eye movement artifacts.

#### Other outcomes

##### Socio-demographic and medical information

Age, civil status, level of education, and annual household income are collected using a self-report questionnaire at baseline (pre-intervention). Medical information on disease stage, treatment protocol, and current medication use are obtained from medical records at baseline (pre-intervention) and at 1-year post-baseline.

##### Psychological health and quality of life

The Hospital Anxiety and Depression Scale [81], Perceived Stress Scale [82], the RAND 36-Item Health Survey 1.0 [83], FACT-Breast Version 4 [84], and FACT-Fatigue [71] are used to assess anxiety and depressive symptoms, perceived stress, health-related quality of life, breast cancer-related quality of life, and cancer-related fatigue, respectively. These are completed by participants at each of the five timepoints.

##### Physical fitness

Aerobic capacity (VO_2_ peak) is measured with a maximal CPET using a metabolic cart (PARVO Medics in Vancouver and VMAX CPET System in Ottawa) performed by trained technicians/respiratory therapists and staff in medically supervised settings. Resting heart rate, measured using electrodes, and resting blood pressure (mmHg), measured in duplicate on the non-surgical side using a blood pressure monitor, are done as precursory steps to ensure participants are able to perform the physical assessments [50]. Anthropometrics include participants’ height (measured with a stadiometer) and weight (measured on a standard scale) while they wear light clothes and no shoes. These outcomes are assessed at baseline (pre-intervention), end of chemotherapy (post-intervention/post-wait period; primary endpoint), 16-weeks post-chemotherapy (first follow-up) for the UC group (optional), and at 1-year post-baseline (second follow-up).

##### Physical activity

Self-reported physical activity behaviour is assessed using a modified version of the Godin Leisure Time Exercise Questionnaire [85]. This is measured at all five study timepoints.

##### Adherence and attendance to the ACTIVATE intervention

Adherence to the supervised exercise prescription and attendance to supervised exercise sessions are tracked by exercise trainers weekly for enrolled participants. Attendance is defined as number of sessions attended compared to number of prescribed sessions. Adherence is defined as number of sessions where the exercise target was achieved for duration and intensity (i.e., percent of HRR). Reasons for missed exercise sessions or non-adherence to the prescribed exercise targets (duration or intensity) are collected by exercise trainers. Participants track their adherence to the home-based exercise prescription throughout the intervention using a logbook. To improve adherence to the ACTIVATE intervention, participants’ parking expenses are covered and exercise sessions are scheduled around their medical appointments, observed holidays, and personal schedules. Also, exercise sessions are offered on weekdays and weekends during regular working hours, as well as early mornings and evenings.

##### Plan to promote participant retention

To help promote participant retention in the trial, participants are provided with a “report card” upon completing the 1-year follow-up assessment. This report card outlines their performance on the neuropsychological test battery (presented in percentiles), body composition, and CPET scores at each timepoint.

### Statistical analyses

Descriptive statistics will be computed for baseline socio-demographic and medical characteristics. Baseline socio-demographic and medical characteristics will be compared between participants who have completed assessments and those who dropped out in order to assess for attrition bias. Characteristics commonly associated with attrition (e.g., age, length of chemotherapy) will be included as covariates in all analyses under the assumption of “missing at random”. Analyses will be on an intent-to-treat basis, with per-protocol analyses considered as exploratory.

The primary outcome (i.e., cognitive functioning as indexed by COGSUM scores) will be analyzed using repeated measures ANOVA with fixed terms for time and a treatment by time interaction (constrained baseline differences). The stratification factors (i.e., study site and menopausal status) as well as factors associated with attrition (e.g., age and length of chemotherapy) will be included as covariates in the analyses. Correlation in repeated measures on the same individual over time will be accounted for by explicitly modeling the covariance matrix, with the best-fitting covariance structure decided using likelihood ratio tests and information criteria. The difference between the EX and UC groups at end of chemotherapy (primary comparison) will be calculated as adjusted least square mean difference in change from baseline, together with 95% confidence intervals. 

To test the effect of timing of the aerobic exercise (i.e., during versus post-chemotherapy) on outcomes at 1-year post-baseline, the models as described above will additionally include the response at 1-year follow-up and the contrast of interest will be the difference in change from baseline to 1-year after the start of chemotherapy. The sustainability of the intervention will be examined in each group by testing the change in response from immediately post-chemotherapy (post-intervention/post-wait period) to 1-year post-baseline, together with a 95% confidence interval.

Changes in secondary outcomes will be analyzed based on repeated measures ANOVAs with fixed terms for time and a treatment by time interaction, and adjusting for the same covariates identified for the primary outcome analysis (e.g., study site, menopausal status, age, length of chemotherapy). Correlation in repeated measures on the same individual over time will be accounted for by explicitly modeling the covariance matrix, with the best-fitting covariance structure decided using likelihood ratio tests and information criteria [86]. The difference between the EX and UC groups post-chemotherapy (primary endpoint) will be calculated as adjusted least square mean difference in change from baseline, together with 95% confidence intervals. All tests will be evaluated at the two-sided 5% level of significance. SAS [87] will be used for all analyses.

### Monitoring

Data monitoring and quality assurance of the ACTIVATE trial is performed by a Data Safety Monitoring Board (DSMB). The DSMB is comprised of three researchers who are independent from the investigators of the ACTIVATE trial and from the funding bodies (i.e., Canadian Cancer Society Research Institute, AVON foundation). The DSMB meets bi-annually, in the absence of study investigators, to review descriptive/interim reports outlining recruitment and enrolment numbers, sample characteristics, primary and secondary outcomes, and any adverse events (assessed and graded using the Common Terminology Criteria for Adverse Events). The primary role of the DSMB is to ensure the proper conduct and safety of the trial and to offer/propose suggestions/modifications to the trial based on data provided in the descriptive/interim reports and any potentially new and/or relevant research in the field. Reports generated by the DSMB are sent to the study investigators as well as to the ethics committee at TOH. Moreover, any decision(s) to discontinue or modify the ACTIVATE intervention for a participant is made by the research team, on a case-by-case basis, if there is concern that the intervention is causing harm.

## Discussion

In Canada, approximately 25,000 women are diagnosed with breast cancer each year [1]. Current research shows that chemotherapy leads to abnormalities in brain functioning and structure, including poorer neurocognitive performance and white matter quality, reductions in volume of brain structure, and abnormal neuronal activation patterns [75, 88–91]. These changes in brain functioning and structure are thought to be the underlying cause of cognitive impairments [89]. Indeed, CRCC is among the most common and distressing symptoms reported by women receiving chemotherapy for breast cancer and research shows that CRCC significantly impacts everyday functioning and quality of life [16, 92, 93]. Women describe CRCC symptoms as frustrating, upsetting, and frightening [16], and many report difficulties and/or an inability to return to their previous occupational, familial, and social activities [16, 92, 93]. Many who do return to their normal activities of daily living do so at the cost of considerable additional mental effort [92, 94]. Women receiving chemotherapy for breast cancer also report frustration and discontent with the response of the medical community to their CRCC, either due to a lack of acknowledgement of their symptoms or to a lack of available evidence-based intervention/treatment strategies [16, 93]. Considering the high prevalence of CRCC in this population and the burden CRCC may place on the healthcare system (e.g., greater demand/utilization of healthcare resources) and economy (e.g., lost work-force productivity [92]), identifying evidence-based management and treatment options for CRCC is critical.

Results from observational and experimental studies suggest that aerobic exercise may be an effective strategy to prevent and/or mitigate CRCC [32–37, 95]. However, many of these studies have methodological limitations related to study design (i.e., observational, lack of comparison group, small sample size, short duration of follow-up). A particular shortcoming of published studies concerns the approach used to assess cognitive functioning (i.e., reliance on self-report measures, assessment of cognitive functioning as a secondary outcome, using a single item or a subscale of a questionnaire assessing fatigue or quality of life [40]). Only two published aerobic exercise RCTs have evaluated cognitive functioning as the primary outcome: one in women receiving chemotherapy for breast cancer [45], the other in women who completed chemotherapy for breast cancer [44]. The ACTIVATE trial is the first RCT powered to detect whether aerobic exercise affects cognitive functioning in women both during and post-chemotherapy. The primary objective of the ACTIVATE trial is to test, in a parallel two-arm RCT, the effect of aerobic exercise during chemotherapy on cognitive functioning compared to aerobic exercise post-chemotherapy. Secondary objectives are to: (1) test the effect of EX compared to UC on global measures of brain structure and functioning, overall global and regional brain network organization, and neural mechanisms underlying attention and working memory, and; (2) assess if the timing of the intervention (i.e., during versus post-chemotherapy) moderates its effects on cognitive functioning.

This manuscript describes the ACTIVATE trial design and all relevant elements of the exercise intervention protocol. The trial is conducted using rigorous methodology (i.e., single blind procedures, experienced staff/exercise trainers, recruitment in two Canadian provinces) and is in accordance with SPIRIT guidelines [46]. Importantly, the ACTIVATE trial aims to determine the nature, timing, and potential progression of CRCC during and following chemotherapy for breast cancer, with the primary aim to evaluate the potential mediating impact of aerobic exercise. Additionally, the ACTIVATE trial addresses limitations of previous exercise trials in oncology on this topic by: (1) recruiting an adequate sample size to detect clinically meaningful changes in cognitive functioning, (2) including both self-report and objective measures of cognitive functioning, and (3) assessing outcomes at five timepoints across a 1-year period. Another notable strength of the ACTIVATE trial is the multi-faceted assessment of the impact of aerobic exercise on brain structure and functioning, with measures of neurocognitive performance, white matter integrity, brain volume, and neuronal activation patterns which underlie cognitive impairments. Given the trial design, it will also be possible to examine whether differences in cognitive functioning and quality of life outcomes are related to the timing of the intervention (i.e., during versus post-chemotherapy), and thus guide the optimal administration of exercise interventions. Finally, attendance and adherence data to the exercise intervention will provide useful information regarding women’s ability and willingness to participate in a progressive, periodized, moderate-to-high intensity aerobic exercise intervention 3 days per week throughout chemotherapy (or shortly thereafter).

In conclusion, aerobic exercise is a promising strategy to prevent and/or reduce CRCC and improve quality of life, and in turn, enhance supportive care for women receiving chemotherapy for breast cancer. Thus, the discoveries from the ACTIVATE trial stand to make significant contributions to the current state of knowledge, including informing the development of future exercise interventions to prevent and/or mitigate CRCC. Indeed, should the ACTIVATE intervention prove to be effective, the ultimate goal will be to implement and disseminate it nationally and internationally.

## Supplementary information

**Additional file 1:** SPIRIT Checklist.

## Data Availability

Not applicable. This manuscript does not contain any data.

## Abbreviations

ACTIVATE: Aerobic exercise and CogniTIVe functioning in women with breAsT cancEr
BESA: Brain electrical source analysis
COGSUM: Composite cognitive summary score
CPET: Cardiopulmonary exercise test
CRCC: Chemotherapy-related cognitive changes
DSMB: Data Safety Monitoring Board
EEG: Electroencephalography
EX: Immediate exercise condition
FACT-Cog: Functional Assessment of Cancer Therapy – Cognitive Function
fMRI: functional magnetic resonance imaging
HRR: Heart rate reserve
MoCA: Montreal Cognitive Assessment
MRI: Magnetic resonance imaging
RCT: Randomized controlled trial
SART: Sustained attention to response task
TOH: The Ottawa Hospital
UC: Usual-care, wait-list control condition
WAIS: Weschler Adult Intelligence Scale
